# Author’s Response to Peer Reviews of “Identifying Safeguards Disabled by Epstein-Barr Virus Infections in Genomes From Patients With Breast Cancer: Chromosomal Bioinformatics Analysis”

**DOI:** 10.2196/69307

**Published:** 2025-01-29

**Authors:** Bernard Friedenson

**Affiliations:** 1 Department of Biochemistry and Medical Genetics, Cancer Center, University of Illinois Chicago, 900 s Ashland, Chicago, IL, 60617, United States, 1 8479124216

**Keywords:** breast, cancer, oncology, ovarian, virus, viral, Epstein-Barr, herpes, bioinformatics, chromosome, gene, genetic, chromosomal, DNA, genomic, BRCA, metastasis, biology

*This is the author’s response to peer-review reports for “Identifying Safeguards Disabled by Epstein-Barr Virus Infections in Genomes From Patients With Breast Cancer: Chromosomal Bioinformatics Analysis.”*

## Round 1 Review

### Anonymous [1]

#### Review Report With Major Revisions for the Paper

*Title: “Herpesvirus infections eliminate safeguards against breast cancer and its metastasis: comparable to hereditary breast cancers”*

#### Summary

*The paper [* *2* *] hypothesizes that Epstein-Barr virus (EBV) infections promote breast cancer by disabling cancer safeguards. It is a bioinformatics analysis of public information from about 2100 breast cancers. The study finds that breast and ovarian cancer breakpoints cluster around EBV-associated cancer breakpoints, suggesting a significant role of EBV in promoting these cancers. The paper also identifies similarities in the molecular and cellular disruptions caused by EBV with those found in hereditary breast cancers.*

#### Major Revisions Needed

##### Clarification of Hypotheses and Objectives

*The hypothesis, while intriguing, needs clearer articulation. Specifically, the connection between EBV and breast cancer needs more explicit theoretical underpinning. Clarify the objectives and expected outcomes of the study at the outset.*

Response: The objectives and expected outcomes of the study were clarified at the outset in the Abstract and Introduction.

##### Methodological Rigor and Data Sources

*While the bioinformatics approach is robust, it would benefit from a more detailed description of the methods and algorithms used. Additionally, the selection criteria for the breast cancer data should be justified more thoroughly to avoid selection bias.*

Response: A more detailed description of the methods and algorithms used has been added in the Methods section (page 6).

##### Statistical Analysis

*The statistical methods used need more comprehensive detailing. For complex analyses, ensure the statistical assumptions and any transformations of data are clearly explained. Include more information on the statistical tests used for hypothesis testing and the justification for their use.*

Response: I included more information on the statistical tests, the justification, and limitations of their use (page 7).

##### Comparative Analysis

*The comparison between hereditary breast cancers and those potentially caused by EBV is insightful. However, a more detailed comparative analysis would strengthen the argument. This could include molecular or genetic profiling comparisons.*

Response: I added a more detailed comparative analysis with results in Figure 2H and Table S2, as described on page 10.

##### Discussion on Contradictory or Supporting Evidence

*The discussion section should address not only the supporting evidence but also any contradictory findings in the literature. This balance is crucial for a nuanced understanding of the subject.*

Response: The paper’s hypothesis more clearly accounts for the absence of demonstrable EBV infection in breast cancer, explaining contradictory results. The other contradictory result posits an imperfect palindrome on chromosome 11. This result is tested on page 13.

##### Implications and Future Research Directions

*The implications of these findings are profound but need clearer articulation. Discuss the potential impact on breast cancer treatment and prevention strategies. Also, outline future research directions, particularly in clinical or experimental studies to confirm these bioinformatics findings.*

Response: I articulated the implications of these finding more clearly with their impact on breast cancer treatment and prevention strategies. I also outlined future research directions with clinical or experimental studies to confirm the bioinformatics findings (Discussion, page 16).

##### References


*Please add more background information about breast cancer (please cite: 1. Cao Y, Efetov S, He M, et al. Updated clinical perspectives and challenges of chimeric antigen receptor-T cell therapy in colorectal cancer and invasive breast cancer. Arch Immunol Ther Exp (Warsz). Aug 11, 2023;71(1):19. [doi: 10.1007/s00005-023-00684-x] [Medline: 37566162]; and 2. Liu Y, Lu S, Sun Y, et al. Deciphering the role of QPCTL in glioma progression and cancer immunotherapy. Front Immunol. Mar 29, 2023;14:1166377. [doi: 10.3389/fimmu.2023.1166377] [Medline: 37063864]).*


Response: I added these references.

### Concluding Remarks

*The paper presents a novel and potentially significant hypothesis linking EBV to breast cancer. However, it requires major revisions to enhance its methodological rigor, clarity, and comprehensiveness. Addressing these concerns will significantly strengthen the manuscript’s impact and contribution to the field.*

### Anonymous [3]

*Dear Author,*

*After a thorough review of the paper titled “Herpesvirus infections eliminate safeguards against breast cancer and its metastasis: comparable to hereditary breast cancers” by Bernard Friedenson, here is the negative feedback and evaluation, along with a recommendation for the inclusion of a specific article in the discussion section.*

#### Negative Feedback and Evaluation

##### Clarity and Scope

*The paper ambitiously attempts to link Epstein-Barr virus (EBV) infections to breast cancer development and metastasis. While the hypothesis is intriguing, the narrative sometimes lacks clarity and could benefit from a more focused scope. The vast amount of data and the complex mechanisms presented can be overwhelming and occasionally detract from the main message.*

Response: I focused the scope in this revision in the Abstract and Introduction.

##### Methodological Concerns

*The reliance on bioinformatics analyses and previously published datasets raises questions about the direct experimental validation of the proposed mechanisms. Although the computational approach is valid, the absence of direct experimental evidence or validation in breast cancer samples limits the strength of the conclusions.*

Response: I explained in the Discussion section that direct experimental evidence or validation has already been done. EBV-infected human mammary epithelial cells produce breast cancer in immunosuppressed mice (page 17).

##### Interpretation of Data

*The interpretation of viral homology and its impact on cancer development is speculative in several sections. The connections made between EBV infections, chromosomal breakpoints, and cancerous mutations rely heavily on correlative data without sufficient causal evidence. A more cautious interpretation of the results, highlighting the need for further experimental validation, would strengthen the manuscript.*

Response: I added more evidence (Figure 2H and Table S2) to the association of EBV infection and cancer development and took greater care throughout to interpret the results more cautiously.

##### Consideration of Alternate Hypotheses

*The paper could benefit from a more balanced discussion of alternative hypotheses explaining the observed data. For instance, the role of other environmental, genetic, or lifestyle factors in breast cancer development is not adequately considered. Acknowledging and discussing these potential confounders would provide a more comprehensive understanding of the complex etiology of breast cancer.*

Response: I explained how EBV relates to alternate hypotheses and exacerbates the effects of other known breast cancer risk factors (page 16).

##### References and Current Literature

*While the paper cites a significant amount of relevant literature, it sometimes overlooks recent studies that could either support or challenge the proposed hypotheses. Incorporating a more current and diverse range of references would enhance the paper’s relevance and credibility.*

Response: I included more information from more current and diverse ranges of references.

### Recommendation for Discussion Inclusion

*To broaden the discussion and contextualize the findings within the broader research landscape, it is recommended to include the following article in the discussion section.*

*Al-Awaida W, Al-Ameer HJ, Sharab A, Akasheh RT. Modulation of wheatgrass (Triticum aestivum Linn) toxicity against breast cancer cell lines by simulated microgravity. Curr Res Toxicol. Sep 19, 2023;5:100127. [doi: 10.1016/j.crtox.2023.100127] [Medline: 37767028]*

*Incorporating this article could provide valuable insights into innovative approaches for studying cancer therapies. Specifically, the effects of simulated microgravity on the efficacy of natural compounds like wheatgrass against breast cancer could open up new avenues for research on the environmental and physical conditions affecting cancer treatment outcomes. Discussing this study would enrich the manuscript by introducing the concept of microgravity as a novel factor influencing cancer cell behavior and therapy resistance, thereby offering a broader perspective on cancer research methodologies and therapeutic strategies.*

Response: I could not find a way to apply and cite this interesting work since it was so far afield from the manuscript.

## Round 2 Review

### Anonymous [3]

#### General Comments

*This paper tests the idea that EBV infections can help cause breast cancer by weakening the body’s defenses against cancer. The study uses bioinformatics to compare chromosome breakpoints in breast cancer to those in cancers known to be caused by EBV. The results show that EBV might play a role in breast cancer by damaging important cell functions.*

#### Specific Comments

##### Major Comments

*The methods section needs more details about how the datasets were chosen and combined.*

Response: More details on how the datasets were chosen have been added.

*The discussion should explain more about how EBV might cause the chromosome breaks and rearrangements seen in breast cancer.*

Response: The discussion includes an expanded explanation about how EBV might cause the chromosome breaks and rearrangements seen in breast cancer.

*More data or references are needed to support the idea that EBV helps breast cancer spread to other parts of the body.*

Response: A new Figure 7 and more data have been added. Additional references have also been added, and the metastasis topic has been clarified and expanded.

##### Minor Comments

*Adding more references would strengthen the sections that talk about how EBV affects breast cancer.*

Response: Many more references have been added.

*Figures and tables should be clearly mentioned in the text to help readers follow the data.*

Response: Figures and tables are now more prominently mentioned in the text.

*Some parts of the manuscript need clearer writing and better organization, especially where complex bioinformatics results are explained.*

Response: I revised the manuscript with clearer writing and better organization, especially where complex bioinformatics results are explained.

*The abstract should be revised to clearly highlight the main findings and why they are important.*

Response: I revised the Abstract to highlight the main findings and why they are important.

*Make sure all abbreviations are defined when they are first used to help readers understand the text better.*

Response: I went through the manuscript to be sure all abbreviations were defined. I also added a glossary containing abbreviations, gene names, and viruses.

## Abbreviations

EBV: Epstein-Barr virus
